# Calendar

**DOI:** 10.1155/S1463924683000401

**Published:** 1983


					Journal of Automatic Chemistry, Volume 5, Number 3 (July-September 1983), page 159
Calendar
Editor's note
Organizers of conferences, seminars, etc.
should send details for inclusion in the
calendar as soon as the relevant infor-
mation is available and not later than three
months before the event. This should be
addressed to 'Journal of Automatic
Chemistry', Taylor & Francis Ltd,
4 John Street, London WCIN 2ET.
August 1983
Computing in Clinical Laboratories
To be held from 24 to 26 August in Breda,
The Netherlands.
Further information from Mr R. C. J.
Galle, Stichting Medische Laboratoria,
Bergschot 69, 4817 PA Breda, The
Netherlands.
American Chemical Society: Annual
Meeting
To be held from 28 August to 2 September
in Washington, D.C.
Further informationfrom ACS, 1155 16th
Street, N.W., Washington, D.C. 20036.
September 1983
Clinical Biochemistry Nearer the Patient
To be held from 5-6 September at the
University of Surrey, Guildford, UK.
Further information from Professor
Vincent Marks, Department of Clinical
Biochemistry, University of Surrey,
Guildford GU2 5XH, UK.
Workshop in Liquid Scintillation Counting
To be held from 5-9 September at Queen
Elizabeth College, London. Organized by
the Radiochemical Methods Group ofthe
Analytical Division, Royal Society of
Chemistry.
Further information from Dr G. Ayrey,
Director of the Isotope Unit, Queen
Elizabeth College (University ofLondon),
Campden Hill, London W8 7AH.
Analysis 83: Microcomputers in the
Laboratory
To be held on 13 and 14 September at the
Sudbury Conference Centre, London.
Further information from Beverly
Humphrey, Scientific Symposia Ltd, 33/35
Bowling Green Lane, London EC1R ODA.
1983 Micromeritics Particle Technology
Seminars
To be held on 19 and 20 September in
Houston, Texas, USA. The seminar is
also being run on 21 and 22 September as
part of Labcon 83 in Rosemont, Illinois,
USA; on 26 and 27 September in San
Francisco, California, USA; 28 and 29
September in Los Angeles, California,
USA; and 5 and 6 October in Atlanta,
Georgia, USA.
Further information from Carol Stancel,
Micromeritics Instrument Corporation,
5680 Goshen Springs Road, Norcross,
Georgia 30093, USA.
13th Philips X-ray Conference
To be held from 19 to 23 September in
Durham, UK.
Further information from Maureen
Courtney at Pye Unicam Ltd, York Street,
Cambridge CB1 2PX, UK.
Columns in High Performance Liquid
Chromatography
To be held from 22-23 September at the
University of Cambridge, UK.
Further information from Mrs Annet
Pullen, Hewlett-Packard Ltd, Analytical
Instrumentation, Nine Mile Road,
Easthampstead, Wokingham, Berkshire
RGl 3LL, UK.
Filtech/Dustex 83; Cost-effectiveness of
Filtration/Separation Equipment and
Processes
Both meetings to be held from 27-29
September at the Cunard International
Hotel, London.
Further information about Filtech/Dustex
83 from Filtech Exhibitions Ltd,
2 Woodstock Road, Croydon CR9 1LB;
details about Cost-effectiveness of
F/S Equipment from Derek Wyllie, The
Filtration Society, 2 Woodstock Road,
Croydon CR9 1LB, UK.
HRGC Forum 83
To be held on 13 September at the
University of Nottingham, UK.
Further informationfrom Charles D. Cook,
Erba Science (UK) Ltd, Headlands
Trading Estate, Swindon, Wiltshire SN2
6JQ, UK.
Course on Fibre Optics for
Instrumentation: Basics, Sensors and
Systems
To be held at UMIST, Manchester, UK
from 27-29 September.
Further information from the Conference
Unit, Sira Ltd, South Hill, Chislehurst,
Kent BR7 5EH, UK.
October 1983
Course on Safety of Electrical
Instrumentation in Potentially
Explosive Atmospheres
To be held from 4 to 6 October at
Cudham Hall, Sevenoaks, UK.
Further information from the Conference
Unit, Sira Ltd, South Hill, Chislehurst,
Kent BR7 5EH, UK.
Analyticon 83 and Laboratory 83
To be held from 12 to 14 October at the
Barbican Centre, London.
Further informationfrom Mr G. C. Young,
SIMA (Scientific Instrument Manu-
facturers' Association of Great Britain),
Leicester House, 8 Leicester Street,
London WC2H 7BN.
Course on Testing Complete Thermal
Imaging Systems and Associated Optical
Materials and Components
To be held from 18-20 October in
Chislehurst, UK.
Further information from the Conference
Unit, Sira Ltd, South Hill, Chislehurst,
Kent BR7 5EH, UK.
November 1983
Practical Reliability Assessment in
Industry
To be held on 10 November at the
Zoological Society, London.
Further information from the Conference
Unit, Sira Ltd, South Hill, Chislehurst,
Kent BR7 5EH, UK.
Second African, Mediterranean and Near
East Conference of Clinical Chemistry
To be held from 20 to 24 November in
Cairo.
Further information from the Congress
Secretariat, Second African, Medi-
terranean and Near East Congress of
Clinical Chemistry, 6 Porter Street, Baker
Street, London WIM 1H2.
See p. 167for information about next
year's conferences.
159

				

